# Reverse Tissue Expansion in Gastroschisis: What to do if the Defect is too large to close after Silo Removal?

**Published:** 2015-07-01

**Authors:** Fadel M Chahine, Bishara Atiyeh

**Affiliations:** Department of Surgery – Division of Plastic and reconstructive Surgery, American University of Beirut Medical Center

**Dear Sir**

We read with interest the case report by Adikibi and O'Toole [1] about what they have named “reverse tissue expansion” for the closure of a large gastroschisis defect. The authors must be commended for this highly ingenious technique that has enabled gradual stretching of the tissues with progressive reduction in the defect size without any damage to the skin edges. Unfortunately, what the authors are describing is not “reverse tissue expansion”. It is in fact stress-relaxation and mechanical creep, a well-established mechanism for skin stretching [2]. Instead of expanding the soft tissues to make available additional skin, we have described few years ago a technique specifically useful for scar revision. To avoid excising a scar then closing the wound primarily under tension, it is possible at sites rich in subcutaneous fat to deflate the tissues by liposuction to relax the skin envelope thus indirectly providing additional skin for scar revision or harvesting of full thickness skin grafts allowing relaxed wound closure. We have called this method “reverse tissue expansion” because in fact this is what it is. For scientific accuracy, we believe that the name “reverse tissue expansion” must be reserved to the technique we have described [3-5]. The authors must refer in describing their method of wound closure to what has already been reported in the literature.

## Footnotes

**Source of Support:** Nil

**Conflict of Interest:** None

